# The Awareness of Females About Risk Factors That Lead to Having a Baby With Congenital Heart Disease in Taif, Saudi Arabia

**DOI:** 10.7759/cureus.40800

**Published:** 2023-06-22

**Authors:** Ahmad S Altuwaireqi, Ahmed F Aljouhani, Abdulaziz B Alghuraibi, Ahmed H Alsuhaymi, Riyadh A Alamrai, Salem M Alzahrani

**Affiliations:** 1 Medicine, Taif University, Taif, SAU; 2 Pediatric Cardiology, Taif University, Taif, SAU

**Keywords:** babies, cardiac, pregnancy, taif, congenital, risk, woman, awareness

## Abstract

Background

There is limited data on the awareness of risk factors associated with congenital heart diseases in Saudi Arabia. This study assesses females’ knowledge of the risk factors that lead to giving birth to a child with congenital heart disease in Taif, Saudi Arabia.

Methodology

A cross-sectional study was done on 254 females. An online questionnaire was used to collect data about the participants’ demographics and their knowledge of risk factors that lead to having a baby with congenital heart disease, including risks such as smoking, drinking alcohol, taking unprescribed medication, exercising, contracting German measles, developing thyroid disease, and not taking vitamins and folic acid, as well as genetic factors such as high blood pressure, diabetes, obesity, consanguineous marriage, advanced maternal age, and eating unhealthy food.

Results

The most common risk factors linked to newborns with congenital heart disorders (CHDs) are alcohol consumption (98.4%), smoking (96%), genetics (86.6%), high blood pressure (82.3%), diabetes (78.4%), and taking medication during pregnancy (74.4%). A little over 73.3% of the participants were aware that risk factors for preterm birth included not taking vitamins and folic acid during pregnancy, obesity (68.9%), contracting German measles while pregnant (68.5%), consanguineous marriage (62.2%), developing thyroid disease during pregnancy (56.7%), and advanced maternal age (50%); 11.4%, 46.1%, and 42.5% of the participants had poor, fair, and good understanding, respectively, of the risk factors for having a baby with congenital cardiac disease. There was no significant correlation between the participants’ demographic characteristics and their levels of awareness.

Conclusion

There is a need for public programs to increase awareness about the risk factors associated with congenital heart diseases.

## Introduction

Congenital heart disorders (CHDs) are defects in the architecture of the heart that may affect venous drainage, cardiac segment septation, and the normal operation of the valve apparatuses [1]. The primary cause of mortality and morbidity, particularly in the first year of life, is CHD. While about 30% of these problems may have related anomalies in other human organs or systems, the majority of these defects appear as isolated anomalies of the heart [1].

About 17.9 per 1000 people worldwide were diagnosed with CHD in 2017, with 19.1 per 1000 males and 16.6 per 1000 females [2]. The most prevalent subtypes of congenital heart disease were ventricular septal defect (VSD) and atrial septal defect (ASD). Globally, researchers discovered that the prevalence of congenital heart disease at birth was highest in Asian countries (9.3/1000 live births) and lowest in African countries (1.9/1000 live births) [3].

A systematic review that included 260 studies found that the birth prevalence of CHD from 1970 to 2017 progressively increased to a maximum of 9.410/1000 in the period 2010-2017 [3]. Another study conducted in Saudi Arabia observed that of the 28646 newborn babies who met the study criteria, 424 (1.48%) had congenital heart conditions [4]. Substantial noncardiac defects were detected in 170 (41.1%) of the 424 newborns with CHD. Among those with severe congenital heart disorders, 91 had trisomy, which is the most prevalent chromosomal aberration [4].

The most prevalent CHD identified was isolated secundum atrial septal abnormality, with a frequency of 4.7/1000 live births [5]. Single ventricular septal defect (VSD) was observed in 2.69/1000 total births, while 7.7% (91/1179) of neonates with birth defects (BD) had severe CHD, which had a frequency of 3.2/1000 births overall. A rate of 11.6/1000 live births led to the diagnosis of less severe CHD in 333 neonates [5].

Of the 1127 patients with CHD in a study conducted at the Madinah Cardiac Center in Saudi Arabia, there were roughly 51.8% male patients and 48.2% female patients, amounting to a male-female ratio of 1.1:1 [6]. A previous study was conducted in Saudi Arabia to assess the demographic and diagnostic details of CHD patients referred to Madinah. It was discovered that 62.6% of the children were between the ages of six and 10 and that cyanotic CHDs were the most common lesions, accounting for 84.8% of all cases [6]. The most prevalent cyanotic congenital cardiac conditions were atrioventricular septal defect (AVSD), coarctation of the aorta (CoA), patent ductus arteriosus (PDA), VSD, and atrial septal defect (ASD) [6]. The most prevalent cyanotic congenital cardiac conditions were tetralogy of Fallot (ToF) (8.7%), transposition of the great arteries (TGA) (1.7%), and truncus arteriosus (1.1%) [6].

A maternal age exceeding 31 years and the low consumption of multivitamins and folic acid during the first trimester are risk factors that have appeared in numerous research studies. Viral infection during the first trimester of pregnancy, such as the rubella virus, is another factor. Congenital cardiac disorders are more common in females with poor obstetric history and those with pregestational diabetes [7].

Obesity and thyroid disorders are frequently linked to ventricular septal abnormalities, hypertension, and other congenital heart problems [8]. Atrial septal defects, truncus arteriosus defects, and secundum arterial septal defects are frequently associated with systemic connective tissue condition, which is also frequently associated with heterotaxy syndrome, alcohol consumption, drug use, and first trimester cigarette smoking [9].

There is limited data on the awareness of risk factors associated with having a baby with congenital heart disease in Saudi Arabia. This study aimed to assess the awareness of females of these risk factors in Taif, Saudi Arabia.

## Materials and methods

Study design, setting, and time

A quantitative, descriptive, cross-sectional study was done in Taif, Saudi Arabia, from May to August 2022.

Study participants

The inclusion criteria were any female between 18 and 50 years of age, married or single, citizen or resident, living in Taif, Saudi Arabia. The exclusion criteria included females over 50 or those less than 18 years of age, as well as females with psychiatric disabilities.

Ethical consideration

The Research Ethics Committee of Taif University reviewed the study, and the committee was accredited by the National Committee for Bioresearch (number: HAO-02-T-105) and considered that the study fulfils the requirements of Taif University, and accordingly, ethical approval was granted.

Data collection

An online questionnaire was distributed as a Microsoft Forms (Microsoft® Corp., Redmond, WA) and published in social media platforms such as Twitter, Instagram, Snapchat, and WhatsApp. The questionnaire collected details of the participants’ demographics and included 14 items to assess their knowledge of risk factors that result in having a baby with congenital heart diseases. These risk factors included risks during pregnancy such as smoking, drinking alcohol, taking medication, exercising, contracting German measles, developing thyroid disease, and not taking vitamins and folic acid, as well as genetic factors such as high blood pressure, diabetes, obesity, consanguineous marriage, advanced maternal age, and eating unhealthy food. The participants were categorized into three levels of knowledge: 1) poor, if they answered less than 50% correctly; 2) fair, if they answered between 50% and 75% correctly; and 3) good, if they answered more than 75% correctly [10].

Data analysis

Data was analyzed using the Statistical Package for Social Sciences (SPSS) version 26 (IBM SPSS Statistics, Armonk, NY). To assess the relationship between variables, qualitative data was expressed as numbers and percentages, and the chi-squared (χ^2^) test was used. Quantitative data was expressed as mean and standard deviation (mean ± SD), and a p-value of <0.05 was considered statistically significant.

## Results

Table 1 shows that 40.9% of the female participant were between 18 and 25 years of age, 52.4% were married, and 71.3% had a university level of education. The majority of the participants (56.75%) were unemployed.

**Table 1:** Distribution of the participants according to their demographic data (number: 254)

Variable	Number (%)
Age (years)	
<18	3 (1.2)
18-25	104 (40.9)
26-45	100 (39.4)
>45	47 (18.5)
Marital status	
Widow	3 (1.2)
Single	113 (44.5)
Married	133 (52.4)
Divorced	5 (2)
Educational level	
Primary	9 (3.5)
Middle	7 (2.8)
Secondary	43 (16.9)
Diploma	2 (0.8)
University	181 (71.3)
Postgraduate	12 (4.7)
Employment	
Housewife	2 (0.8)
Student	7 (2.8)
Not employed	144 (56.7)
Retired	11 (4.3)
Employed	90 (35.4)

**Figure 1:**
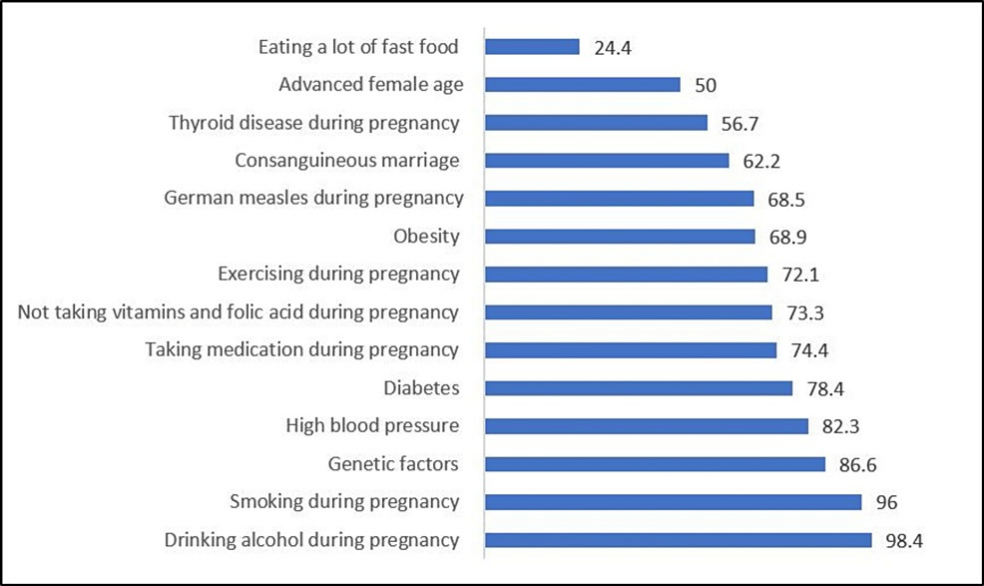
Percentage distribution of the participants according to the number of correctly answered questions on risk factors associated with newborns with congenital heart disease (number: 254)

Table 2 and Figure 1 show the participants’ responses to knowledge items about risk factors that lead to having a baby with congenital heart disease. The most common risk factors that were identified correctly were drinking alcohol during pregnancy (98.4%), smoking during pregnancy (96%), genetic factors (86.6%), high blood pressure (82.3%), diabetes (78.4%), and taking medication during pregnancy (74.4%). At the same time, more than half of the participants were aware of the risks associated with not taking vitamins and folic acid during pregnancy (73.3%), obesity (68.9%), contracting German measles during pregnancy (68.5%), consanguineous marriage (62.2%), developing thyroid disease during pregnancy (56.7%), and advanced maternal age (50%). For the two items stating that exercising during pregnancy and eating a lot of unhealthy food were not risk factors, 72.1% correctly disagreed on the former and 24.4% on the latter.

**Table 2:** Distribution of the participants according to their responses to knowledge items about risk factors associated with having a baby with congenital heart disease (number: 254)

Variable	Strongly disagree	Disagree	Neutral	Agree	Strongly agree
	Number (%)	Number (%)	Number (%)	Number (%)	Number (%)
Do you think that advanced age in females is a risk factor?	18 (7.1)	48 (18.9)	61 (24)	82 (32.3)	45 (17.7)
Do you think that exercising during pregnancy is a risk factor?	39 (15.4)	144 (56.7)	46 (18.1)	24 (9.4)	1 (0.4)
Do you think diabetes is a risk factor?	5 (2)	3 (5.1)	37 (14.6)	150 (59.1)	49 (19.3)
Do you think high blood pressure is a risk factor?	0 (0.0)	10 (3.9)	35 (13.8)	145 (57.1)	64 (25.2)
Do you think obesity is a risk factor?	1 (0.4)	9 (11.4)	49 (19.3)	115 (45.3)	60 (23.6)
Do you think smoking during pregnancy is a risk factor?	0 (0.0)	3 (1.2)	7 (2.8)	74 (29.1)	170 (66.9)
Do you think drinking alcohol during pregnancy is a risk factor?	0 (0.0)	1 3(0.4)	3 (1.2)	59 (23.2)	191 (75.2)
Do you think that not taking vitamins and folic acid during pregnancy is a risk factor?	5 (2)	19 (7.5)	44 (17.3)	99 (39)	87 (34.3)
Do you think that thyroid disease during pregnancy is a risk factor?	3 (1.2)	27 (10.6)	80 (31.5)	100 (39.4)	44 (17.3)
Do you think having German measles during pregnancy is a risk factor?	1 (0.4)	4 (5.5)	65 (25.6)	111 (43.7)	63 (24.8)
Do you think that eating a lot of fast food is a risk factor?	8 (3.1)	54 (21.3)	70 (27.6)	86 (34.3)	35 (13.8)
Do you think that taking medication during pregnancy is a risk factor?	1 (0.4)	14 (5.5)	50 (19.7)	116 (45.7)	73 (28.7)
Do you think that consanguineous marriage is a risk factor?	9 (3.5)	23 (9.1)	64 (25.2)	86 (33.9)	72 (28.3)
Do you think genetic conditions are risk factors?	2 (0.8)	7 (2.8)	25 (9.8)	116 (45.7)	104 (40.9)

**Figure 2:**
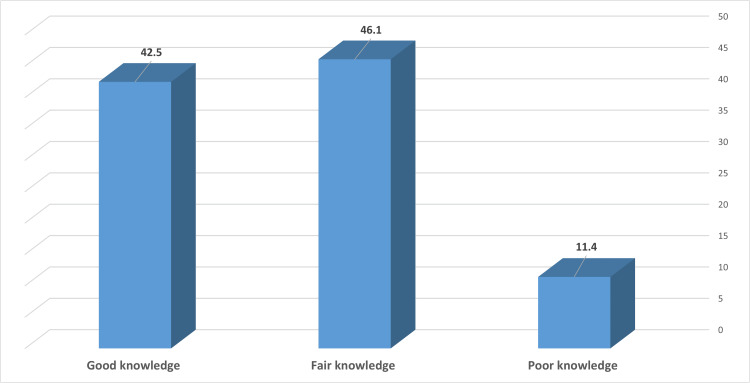
Percentage distribution of the participants according to their knowledge level of risk factors associated with having a baby with congenital heart disease (number: 254)

The prevalence of poor, fair, and good knowledge levels about risk factors associated with having a baby with congenital heart disease among studied females was 11.4%, 46.1%, and 42.5%, respectively (Figure 2).

Table 3 demonstrates that a non-significant relationship was found between the participants’ knowledge level about risk factors in having a baby with congenital heart disease and their demographic characteristics (p≥0.05).

**Table 3:** Relationship between participants’ knowledge level of risk factors associated with having a baby with congenital heart disease and their demographic characteristics (number: 254) χ^2^: chi-squared

Variable	Knowledge level			χ^2^ (degree of freedom {df})	P-value
	Poor: number (%)	Fair: number (%)	Good: number (%)		
Age (years)					
<18	1 (3.4)	2 (1.7)	0 (0.0)	5.13 (6)	0.527
18-25	14 (48.3)	49 (41.9)	41 (38)		
26-45	10 (34.5)	47 (40.5)	43 (39.8)		
>45	4 (13.8)	19 (16.2)	24 (22.2)		
Marital status					
Widow	0 (0.0)	2 (1.7)	1 (0.9)	8.51 (6)	0.203
Single	16 (55.2)	58 (49.6)	39 (36.1)		
Married	13 (44.8)	56 (47.9)	64 (59.3)		
Divorced	0 (0.0)	1 (0.9)	4 (3.7)		
Educational level					
Primary	3 (10.3)	5 (4.3)	1 (0.9)	10.59 (10)	0.39
Middle	1 (3.4)	4 (3.4)	2 (1.9)		
Secondary	4 (13.8)	21 (17.9)	18 (16.7)		
Diploma	0 (0.0)	1 (0.9)	1 (0.9)		
University	21 (72.4)	82 (70.1)	78 (72.2)		
Postgraduate	0 (0.0)	4 (3.4)	8 (7.4)		
Employment					
Housewife	0 (0.0)	1 (0.9)	1 (0.9)	13.38 (8)	0.099
Student	0 (0.0)	5 (4.3)	2 (1.9)		
Not employed	24 (82.8)	67 (57.3)	53 (49.1)		
Retired	0 (0.0)	6 (5.1)	5 (4.6)		
Employed	5 (17.2)	38 (32.5)	47 (43.5)		

## Discussion

This study aimed to determine females’ awareness of the risk factors associated with having a baby with congenital heart disease in Taif, Saudi Arabia.

The study found that the most commonly known risk factors for having a baby with congenital heart disease were drinking alcohol during pregnancy (98.4%) and smoking during pregnancy (96%). This is in agreement with a previous study done in Iran, where almost all of the participants identified drinking alcohol and smoking as risk factors for congenital abnormalities [11].

Several research studies, including ours, established a connection between maternal smoking and congenital abnormalities [12,13]. The etiology of the majority of CHDs is still a mystery. Sullivan et al. [14], however, discuss the findings of a population-based study in which they examined the potential link between maternal periconceptional smoking and the prevalence of CHDs in live infants.

Smoking is prohibited for females in the Kingdom of Saudi Arabia (KSA) due to cultural norms, so smoking among females is still extremely low, even though the modernization of females, which enables them to work outside the home, is making some cultural barriers obsolete. Alcohol consumption is prohibited in the Islamic religion due to its detrimental effects on society. Perhaps, this is one of the key factors contributing to females abstaining from alcohol consumption as they are aware of the risks involved. Scientific literature and articles have reported a connection between alcohol use and congenital abnormalities [13,15].

In a study conducted in Ethiopia, almost half of the participants’ beliefs about the causes of congenital malformations (CMs) were related to sin, contraception pills, unprescribed drug/medication use, and fertilizers. In addition, most of the participants were unaware of the effect of smoking on the development of the embryo, which led to congenital abnormalities [16].

In the study, 50% of the participants believed that advanced maternal age is a risk factor. Numerous research studies have discovered a link between maternal age and congenital abnormalities [17,18].

About 74% of the participants concurred that taking non-prescribed medicines while pregnant poses a danger. In a survey conducted in Iran, 88% of the participants were aware of the negative effects of taking non-prescribed medication during pregnancy, further demonstrating a high level of awareness of these issues [11]. On the other hand, an older study conducted in Nigeria discovered that a significant segment of the respondents was unaware that advanced maternal age and the lack of rubella vaccination and folic acid supplementation prior to conception were risk factors in delivering children with congenital abnormalities [19].

Recently, health centers have been advised not to take unprescribed prescriptions. This is possibly why the participants in the study were aware of this [11]. A little over 62% of the study’s participants agreed that consanguinity is a risk factor for congenital abnormalities, a result that is consistent with a prior Iranian study in which 82% of respondents thought the same [11]. The outcomes are consistent with those of earlier investigations, which demonstrated a higher incidence of severe birth abnormalities in children of consanguine parents [20,21]. This is in contrast to the studies of Zhang et al. [22] and Karbasi et al. [23], who found congenital abnormalities and consanguinity to not be related. On the other hand, other studies revealed that genetic counselling prior to conception could be very beneficial for prevention [23].

The current study found a higher level of knowledge regarding CHD risk factors in Taif, Saudi Arabia, than previously reported in Ghana [24] and Kenya [25], where the majority of the participants were unaware of congenital malformations (CMs) and their causes [24,25]. This suggests that different communities held similar views and thought that CMs were a strange phenomenon. This might be a result of different educational backgrounds, the lack of knowledge regarding CMs, and the influences of various religions, cultures, and cultural standards.

Thyroid disorders during pregnancy (56.7%), German measles (68.5%), not taking vitamins or folic acid (73.3%), and diabetes (78.4%) were risk factors among the individuals in the study. Certain genetic disorders and birth defects can be avoided, particularly through interventions such as folic acid supplementation before pregnancy [26], the iodination of food items [27], immunization with the rubella vaccine [28], and prompt diagnosis and treatment of preexisting health conditions before pregnancy. It takes an educated mother to be aware of all these interventions and to know when and how to seek help at a health facility [29].

According to this study, 11.4%, 46.1%, and 42.5% of the female participants had poor, fair, or good understanding, respectively, of the risk factors associated with congenital cardiac disease. A similar outcome was seen in a Nigerian study, where only 50% of the participants had good awareness of the causes and effects of genetic illnesses and congenital defects [19]. Another study done in Nigeria, however, revealed a lower degree of knowledge, with only 25% of mothers exhibiting a knowledge of factors related to birth abnormalities [30].

This study discovered a non-significant association between the participants’ demographic characteristics and their level of knowledge of risk factors for having a baby with congenital heart disease. Similar research findings were made by Bello et al., who found no association between the participants’ general knowledge of congenital abnormalities and their age or educational qualifications. Their results did not match with the results of this study [24]. This data therefore implies that motivation in learning is more likely than age to result in better health education.

A different outcome can be found in a prior Iranian study, where there was a substantial correlation between age and educational level. The participants who had completed high school and a university degree in this Iranian study scored the highest overall, in terms of the knowledge of the illness. Additionally, a knowledge of the illness was observed to be the lowest overall among people aged 41-50 years, while the participants between the ages of 21 and 30 years had the best understanding of the risk variables [11]. However, in a study conducted in Nigeria, an age factor of 30 years and above was linked to higher knowledge levels [19].

In the prior Nigerian study, it was shown that there was a correlation between the working class, particularly the upper/middle socio-economic strata, and the awareness of birth abnormalities [30]. This was attributed to the working class’s superior access to informational resources when compared to the unemployed sector.

Limitation

A limitation of this work is the cross-sectional study design that could reveal the associations between studied variables but not the causal relationships. Future research could investigate the causal relationships between the variables studied here.

## Conclusions

This study found that the most common risk factors for congenital heart diseases were drinking alcohol during pregnancy (98.4%), smoking during pregnancy (96%), genetic factors (86.6%), high blood pressure (82.3%), diabetes (78.4%), and medication during pregnancy (74.4%). About 73.3% of the participants were aware of the risk factors such as not taking vitamins and folic acid during pregnancy (73.3%), obesity (68.9%), German measles infection during pregnancy (68.5%), consanguineous marriage (62.2%), developing thyroid disease during pregnancy (56.7%), and advanced maternal age (50%). Of the participants, 11.4%, 46.1%, and 42.5% had poor, fair, and good knowledge levels, respectively, of risk factors associated with congenital heart disease. A non-significant relationship was found between the participants’ knowledge levels and their demographic characteristics. Both governmental and nongovernmental health institutions should foster an atmosphere that allows medical professionals to educate couples, expectant mothers, and the community at large about preventable risk factors for congenital malformations.
